# Capital Punishments, Psychologically Considered

**Published:** 1850-01-01

**Authors:** 


### Art. VII.—CAPITAL PUNISHMENTS:

### PSYCHOLOGICALLY CONSIDERED.

The state-policy of capital punishments, whether for high political misdemeanors or social offences, has long been contested; and the discussion, it would appear, is now revived by the execution of two wretched criminal— Manning and his wife—at Horsemonger-lane gaol, on the 13th of last November, 1849. One of our most popular novelists, Mr. Charles Dickens, addressed a letter to the daily newspapers, describing the demoralizing aspect of the scene, and calling upon the Secretary of State for the Home Department to fulfil his promise, made in the House of Commons, that he would take into consideration the expediency of some less public mode of carrying the law into effect. "I believe that a sight so inconceivably awful as the wickedness and levity of the immense crowd collected at that execution could be imagined by no man, and could be presented in no heathen land under the sun. The horrors of the gibbet and of the crime faded in my mind before the atrocious bearing, looks, and language of the assembled spectators. * * * When the sun rose brightly as it did, it gilded thousands upon thousands of upturned faces so inexpressibly odious in their brutal mirth or callousness, that a man had cause to feel ashamed of the shape he wore, and to shrink from himself as fashioned in the image of the devil. When the two miserable creatures who attracted all this ghastly sight about them were turned quivering into the air, there was no more emotion, no more pity, no more thought that two immortal souls had gone to judgment, no more restraint in any of the previous obscenities, than if the name of Christ had never been heard in this world, and there were no belief among men but that they perished like the beasts. I am solemnly convinced that nothing that ingenuity could devise to be done in this city in the same compass of time could work such ruin as one public execution, and I stand astounded and appalled by the wickedness it exhibits."* Another accomplished author, whose name is well known in the higher walks of dramatic literature—R. H. Horne—has also put forth a pungent satire in Dean Swift's best style, under the title of "Murder Heroes," pointing out the impolicy of exciting the sympathy of the multitude in behalf of common felons, and thereby exciting a morbid curiosity and interest respecting

> "All they said and did;
> How they were dressed;
> What they ate and drank;
> And with what sort of step they
> Ascended the scaffold."†

The moral is here obvious; but it is to be remembered that the publicity of capital punishments is supposed to be absolutely necessary for their efficacy, inasmuch as such punishments are not intended, as Blackstone observes, "by way of atonement or expiation for the crime committed, for that must be left to the just determination of the Supreme Being, but as a precaution against future offences of the same kind. This is effected three ways: either by

* Letter of Mr. Charles Dickens to the daily newspapers, Nov. 13, 1349.

† Murder Heroes, and the Diseased Drama of their Crime, Sentence, and Execution. By R. H. Horne. Kent and Richards, 1849.

the amendment of the offender himself, for which purpose all corporal punishments, fines, and temporary exile, or imprisonment, are inflicted; or by deterring others, by the dread of his example, from offending in the like way, 'ut pœna (as Tully expresses it) ad paucos, metus ad omnes, perveniat,' which gives rise to all ignominious punishments, and to *such executions of justice as are open and public;* or, lastly, by depriving the party injuring of the power to do future mischief, which is effected by either putting him to death, or condemning him to perpetual confinement, slavery, or exile."* We believe that in all countries throughout the world, whatever may be their degrees of civilization, the crime of murder is followed by the execution or death of the murderer. "He that sheddeth man's blood, by man shall his blood be shed," was the Jewish law;† and although, under the Christian dispensation, the severity of the penal code was greatly relaxed, inasmuch as, without any special divine interposition, capital punishments were no longer inflicted upon such criminals as idolaters (Exodus xxii. 20), blasphemers (Leviticus xiv. 15, 16), necromancers and witches (Leviticus xx. 27), adulterers (Exodus xxi. 6), and many other offences. The sentence of death for murder was never, even by our Saviour, rescinded. Here, however, it is to be observed that there are two points at issue: the first is the abolition of capital punishments; the second is the expediency of the execution taking place privately. The number of offences punishable by death, according to our statute books, was, until a few years ago, very considerable; but in accordance with that benign spirit of toleration which has gradually infused more enlarged and enlightened views into the principles of legislation, the penalty of death is now affixed to a very small number of offences, and is rarely carried into effect, excepting in cases of murder. Unhappily, however, for the cause of those who desire the total abolition of capital punishment, the statistics of crime irrefragably prove that—even making every allowance for the increase of population—the diminution in capital punishments has been followed by a very large increase both in the offences previously punished by death, and in the general amount of crimes.‡ This is not all. While it appears that those offences for which the punishment of death has been abolished have increased in number, those crimes which are still so punished have not increased in anything

* Blackstone's Commentaries. Edited by Edward Christian. Book IV. chap. 1, vol. iv. p. 10. London, 1830.

† Genesis ix. 6.

‡ Law Magazine, N. S. Vol. iv. p. 228. 1846. Ibid. vol. x. p. 204. 1809.

like the same proportion; hence, the only rational inference is, "that the fear of capital punishments does deter from the commission of those crimes to which it is attached."* Very lately, Mr. Justice Coleridge, in accordance with these facts, stated from the bench that it grieved him to find that "crimes from which capital punishments had been taken away had increased more in proportion than any other." It is also important to remark that Sir Samuel Romilly, who, adopting the principles of Montesquieu and Beccaria, devoted so many years of his valuable life to advocating a mitigation of the penal code, emphatically observed, "I confess it appears absolutely impossible to omit death in the catalogue of human punishments."† Without entering further into the consideration of this important part of the subject, which the statistical tables of Mr. Redgrave, laid before Parliament by command of her Majesty, have thrown so much light upon, we shall proceed—as coming more immediately within our province—to consider capital punishments in their psychological relations; that is to say, the effect of the sentence of death upon the mind of the condemned criminal, and the effect of public executions upon the mind of the gazing multitude.

We must here premise that the constitution of the human mind differs as much as the constitution of the human body in different individuals; and as the body is endowed with different temperaments, which greatly modify the affections to which it is liable, so the human mind possesses, in every individual, its own peculiar idiosyncrasy, which modifies, in like manner, its impressions and manifestations, giving more or less energy and direction to all its faculties. We must also take into consideration the influence of education, habits of life, and religion; with a variety of exciting causes which may inspire the mind with a preternatural energy in the hour of trial, or which may overwhelm and paralyze its powers, and destroy even the consciousness of existence.

When the amiable and accomplished Lady Jane Grey ascended the scaffold, with saint-like demeanour she displayed an example of calmness and self-possession—a sense of piety and resignation unparalleled in the annals of either ancient or modern history. Her conduct on the scaffold is thus described in the "State Trials":—"She was attended by Dr. Feckenham (formerly Abbot of Westminster), but was observed not to give much heed to his discourses, keeping her eyes constantly fixed on a book of prayers which she

* Ibid. vol. iv. p. 231.

† Life and Correspondence of Sir Samuel Romilly. Edited by his son. Vol. i. p. 29.

held in her hand. After some short recollection, she saluted those who were present,* with a countenance perfectly composed; then, taking leave of Dr. Feckenham, she said, 'God will abundantly requite you, good sir, for your humanity to me, though your discourses gave me more uneasiness than all the terrors of my approaching death.' She next addressed herself to the spectators in a plain short speech; then kneeling down, she said the *Miserere*, in English; after which she stood up and gave her women (Mrs. Elizabeth Tilnes and Mrs. Helen) her gloves and her handkerchief, and to the lieutenant of the Tower (whom Heylin calls Sir John Page, but Hollinshed, Bridges) her prayer-book. When she untied her gown, the executioner offered to assist her, but she desired him to let her alone; and turning to her women, they undressed and gave her a handkerchief to wind about her eyes. The executioner, kneeling, desired her pardon, to which she answered, 'Most willingly.' He desiring her to stand upon the straw, which bringing her within sight of the block, she said, 'I pray despatch me quickly,' adding presently after, 'Will you take it off before I lay me down?' The executioner said, 'No, madam.' Upon this, the handkerchief being bound close over her eyes, she began to feel for the block, to which she was guided by one of the spectators; when she felt it, she stretched herself forward, and said, 'Lord, into thy hands I commend my spirit;' and immediately, at one stroke, her head was severed from her body."†

The calmness and complacency with which this august lady met her death, and which inspired her, in her prison solitude, to write letters breathing the "most sublime sentiments of piety," can only be accounted for by considering the ascendancy which her religious sentiments, and we may say her philosophical principles, had acquired over her feelings; for although only seventeen years of age, the "Phædo" of Plato was one of her favourite books, which she read in Greek "with as much delight (says Roger Ascham) as some gentlemen would read a merry tale in Boccacio."‡ The sight of her

* The Court, it is said, had at one time taken a resolution that she should be beheaded on the same scaffold with her husband; but considering how much they were both pitied, and how generally Lady Jane Grey was beloved, and to prevent the popular commotion which such a spectacle might excite, it was determined that, after he had been executed on Tower Hill, in the presence of an immense multitude, she, on account of her Royal descent, should be spared the ignominy of a public execution.

† State Trials, vol. i. 1554, p. 773. Hansard. 1809.

‡ Somers' Tracts, 4th col. vol. i. 174.

husband's headless body, which she saw carried past her prison window, streaming with blood, must have reconciled her to meet a similar fate without a murmur. Hence, when the queen, on that fatal morning, sent them permission to take a last farewell of each other, Lady Jane refused the indulgence, saying that "in a few hours she should meet her dear lord in heaven."

> "So through the cloud of death her spirit pass'd
> Into that pure and unknown world of love
> Where injury cannot come."*

We should err, however, greatly, if we presumed that an indifference to life is at all a common condition of the human mind, even under sentence of death; the more natural effect of such judgment, particularly upon the young, is to overwhelm and disintegrate the system mentally and physiologically. "The very thoughts of death," observes Lady Gethin, "disturb one's reason; and although persons may have many excellent qualities, yet may they have the weakness of not being able to command their sentiments."† "Men fear death," observes Lord Bacon, "as children fear going into the dark."‡ Hence, in that very painful and very remarkable tragedy, "The Cenci," when Beatrice, whose lovely portrait may still be seen in the Colonna Palace at Rome, as Guido himself painted it during her confinement in prison, was condemned to death, she exclaimed, in a paroxysm of mental agony—

> "Oh!
> My God! can it be possible I have
> To die so suddenly? So young to go
> Under the obscure, cold, rotting, wormy ground,
> To be nailed down into a narrow place;
> To see no more sweet sunshine; hear no more
> Blithe voice of living thing; muse not again
> Upon familiar thoughts; sad, yet thus lost,
> How fearful! To be nothing, or to be—
> What? O where am I? Let me not go mad!
> Sweet Heaven, forgive weak thoughts!"§

The feelings of apprehension and dismay here expressed are perfectly in accordance with nature, and inherent in our instinctive love of life and desire of self-preservation. It remains, therefore, for the psychologist to observe under what circumstances this elementary law of humanity becomes modified, changed, or perverted. Besides

* Wordsworth's Excursion.

† Reliquiæ Gethianæ, by Lady Gethin—a very rare volume cited by Israeli.

‡ Lord Bacon. Essay on Death.

§ Shelley's Cenci, Act v. sc. 4.

the idiosyncracy, which we have just described, as peculiar to every individual mind, extraneous influences operating as proximate causes on our habitual modes of thought and feeling must be taken into consideration. In the case of Lady Jane Grey, we find a degree of mental resignation, so pure and so devout as to be unsullied by the slightest shadow of affectation. But many other noble sufferers have conducted themselves even on the scaffold with an affected levity and a certain air of ostentation, as if they fancied that posterity would be impressed with "all they said and did" in the last publicly exhibited moments of life. When Sir Thomas More was brought out of the Tower to be beheaded, his beard was long, his face pale and thin, he was weak, and seemed ready to fall; but as he mounted the platform, he said merrily to the lieutenant who assisted him, "Pray see me safe up, and as to my coming down let me shift for myself." Afterwards, when he laid his head upon the block, he bid the executioner "stay till he had put his beard aside, for that had committed no offence."*

When Sir Walter Raleigh was taken out of his bed in a fit of fever, and unexpectedly hurried, not to his trial, but to a sentence of death, he pleaded his cause with "a voice grown weak by sickness, and an ague he had at the time." He used every means to avert his fate; for he valued his life, and would not willingly part with it; but when sentence of death was inexorably passed upon him, and the judge ended with saying, "execution is granted," the heroic sage felt as if listening to fame in the voice of death; "and thereupon entreated permission to speak freely at his farewell, to satisfy the world that he was ever loyal to the king and a true lover of the commonwealth." On his return to prison, while some were deploring his fate, he observed that "the world itself is but a large prison, out of which some are daily selected for execution." The last night of his existence was occupied by writing what the letter-writer calls "A Remembrancer to be left with his Lady," to acquaint the world with his sentiments, should he be denied their delivery from the scaffold, as he had been at the bar of the King's Bench. His lady visited him that night, and amidst her tears acquainted him that she had obtained the favour of disposing of his body; to which he answered smiling, "It is well, Bess, that thou mayst dispose of that dead thou hadst not always the disposing of when it was alive." * * * "It is peculiar in the fate of Raleigh, that having before suffered a long imprisonment, with an expectation of a public death, *his mind had been accustomed to its contemplation, and had often dwelt on the event now*

* "State Trials," 1535. Vol. i. p. 395.

*passing.*" This presentiment—this prefiguring in the mind's eye the details of the last sad scene of all, must not be passed unnoticed herein, because the moral we have set out with hereby becomes more clearly developed. When the fatal hour arrived, "his dress, as was usual with him, was elegant, if not rich." Oldys describes it, and mentions that "he had a wrought nightcap under his hat; he wore a ruff band, a black wrought velvet nightgown over a hair coloured satin doublet, and a black wrought waistcoat; black cut taffety breeches, and ash-coloured silk stockings." He had previously, it appears, on the morning of his death, smoked as usual his favourite tobacco; and when they brought him a cup of excellent sack, being asked how he liked it, Raleigh answered, "As the fellow that, drinking of St. Giles's bowl, as he went to Tyburn, said, 'that was good drink if a man might tarry by it.'" We further read, that after taking off his gown, he called to the headsman to show him the axe, which not being instantly done, he repeated, "I prithee let me see it—dost thou think that I am afraid of it?" He then passed the edge lightly over his finger, and smiling, observed to the sheriff, "This is sharp medicine that will cure all sorrows." When he began to fit himself for the block, he first laid himself down to try how the block fitted him. After rising up, the executioner knelt down to ask his forgiveness, which Raleigh with an embrace gave; but entreated him not to strike until he gave a token by lifting up his hand. When he laid his head down upon the block, the executioner desired him to lay his face "towards the east." "It was no great matter (answered Raleigh) which way a man's head stood, so that the heart lay right." But these were not his last words. He was once more to speak in this world with the same intrepidity he had lived in it; for having lain some minutes on the block in prayer, the executioner, either unmindful or in fear, failed to strike; and Raleigh, after once more or twice putting forth his hands, was compelled to ask him, "Why dost thou not strike? Strike home!" In two blows he was beheaded, but from the first his body never shrank from the spot by any discomposure of his posture, which was like his mind, immoveable." *

We can perfectly understand how the mind of a great statesman can be wrought up to this state of intrepidity. "'Doth any indecent fear betray me?' asked the Earl of Strafford, when he mounted the scaffold. 'Never did I throw off my clothes with greater freedom and content than on this preparation for my grave. That stock (pointing to the block) must be my pillow; here shall I rest from

* Narrative of the Last Hours of Sir Walter Raleigh, in D'Israeli's "Curiosities of Literature." Vol. iii. p. 142, *et seq.* Moxon. 1849.

all my labours. No thoughts of envy, no dreams of treason, jealousies, or cares for the king, the state, or myself, shall interrupt this easy sleep.' After this, going to take off his doublet, he reiterated, 'Thank God, I am not afraid of death, but do as cheerfully take off my doublet at this time as ever I did going to bed.' Then he put off his doublet, wound up his hair with his hands, and put on a white cap. After kneeling in prayer, he said to the executioner that he would first lay his head down to try the fitness of the block, and take it up again before he would lay it down for good and all. And so he did. Presently laying down his head upon the block, he stretched out his hands, and the executioner struck off his head at one blow."*

In like manner, our weak and vacillating unfortunate monarch, Charles I., after his prolonged and anxious trials, conducted himself during the last moments of his life with the greatest firmness and dignity. The night previous to execution, he slept soundly for four hours, and awoke near two hours before daylight; and calling to Mr. Herbert, who lay by his bedside, bid him rise, "for," said the king, "I will get up, as I have a great work to do this day. Herbert! this is my second marriage-day. *I would be as trim to-day as may be;* for before night I hope to be espoused to my blessed Redeemer." He then appointed what clothes he would wear; and says he, "Let me have a shirt on more than ordinary, by reason the season is so sharp as probably may make me shake, *which some observers will imagine proceeds from fear; I would have no such imputation; I fear not death; death is not terrible to me; I bless my God I am prepared.*" When his devotions were ended, he was conducted on foot from St. James's to Whitehall by a regiment of foot soldiers; the Bishop of London on one hand, and Colonel Tomlinson on the other, both being bareheaded. The guards marching at a slow pace, he bade them walk faster, saying that he "now went before them to strive for a heavenly crown, with less solicitude than he had often encouraged his soldiers to fight for an earthly diadem." Upon the scaffold, the same presence of mind was exhibited in his conversation with the Bishop. Looking to the block, he said to the executioner, "You must set that fast." "It is fast," answered the executioner. "Then," rejoined the king, "when I put out my hands this way (stretching them out) then——" after that, having said two or three words to himself, as he stood with his hands and eyes lift up, immediately stooping down he laid his neck upon the block, and then the executioner again putting his hair under his

* "State Trials," 1627. 1640. Vol. iii. 1522.

cap, the king thinking he had been going to strike, said, "Stay for the sign." "Yes, I will," answered the executioner, "an't please your majesty." After a little pause, the king stretched forth his hands, and the executioner at one blow severed his head from his body.*

The ceremonial observed at these state executions deserves attention. It will be observed that the sufferers invariably took particular pains to dress themselves with care for the occasion, some even with unusual ostentation.† When the fatal day arrived for the execution of Mary Queen of Scots, we are told "that she dressed herself as gorgeously as she was wont to do on festival days."‡ It appears also to have been a practice enjoined by confessors for such supposed criminals, to pass an eulogium upon the monarch before submitting to their fate. In the case of Anna Boleyn, she prayed heartily for the king, and "called him a most merciful and gentle prince," adding that "he had always been a good, gentle, sovereign lord"—words which were probably dictated to her by the cautious Cranmer for the sake of her daughter.§ The executioner, before striking the fatal blow, always asked and was granted forgiveness by the state criminal.

"The common executioner,
Whose heart th' accustomed sight of death makes hard,
Falls not the axe upon the humbled neck,
But first begs pardon."||

We do not, however, in all cases, find that spirit of resignation which characterized the conduct of the above illustrious personages. When Margaret, Countess of Salisbury—the mother of Cardinal Pole, seventy years of age—was conducted to the scaffold, animated by the indomitable spirit of her race, she refused, when the executioner desired her to lay her head upon the block, saying that her head had never committed treason ; and that "if he would have it, he must fetch it as he could;" thus saying, she ran about the scaffold in defiance of him, shaking her grey locks at him, and was compelled by force alone to lay her neck upon the block. Even when the blow was about to be inflicted, she exclaimed, "Blessed are they who suffer *persecution* for righteousness' sake."¶ So perished the last

* "State Trials." Vol. iv. 1142.

† It is a curious fact that suicides, particularly females, generally dress themselves in their best clothes before committing the fatal deed.

‡ "State Trials." Vol. i. 1210.

§ Ellis. Vol. ii. p. 66. || Shakspeare.

¶ "Thomson's Memoirs of the Court of Henry VIII. Vol. ii. p. 513. London. 1826.

of the Plantagenets—a victim to the jealousy and cruelty of one the most inexorable tyrants that ever disgraced a throne.

We now proceed to consider the effect of the sentence of death upon minds of a very different order — offenders who incur this penalty for social crimes. Here we may at once predicate that difference of education, rank, and a variety of circumstances, will give rise to very opposite manifestations of thought and feeling; and here, again, we must consider the constitution and idiosyncracy peculiar to different minds. In the age to which we have referred there was no ignominy, and scarcely any dishonour, in dying upon the scaffold. But the axe and the rope present a very different type of punishment. To be hung, like a dog, by the neck, and left for an hour swinging to and fro in the wind, is a spectacle too degrading for humanity; and certainly to a mind of any sensibility must materially aggravate all the horrors of premature death. In the year 1777, Dr. Dodd was found guilty of forgery, and uttering as true a counterfeit bond, purporting to be the bond of the Earl of Chesterfield, to whom he was chaplain, for the sum of 4200*l.* He was condemned to be hanged. Upon the sentence being pronounced by the recorder, the miserable divine sank down overwhelmed with agony, and was taken out of the court uttering the most dismal lamentations. "I feel," he exclaimed, when asked why judgment of death should not be passed upon him, "the natural horror of a violent death, and the universal dread of untimely dissolution. * * The gloom and confusion of a prison, the anxiety of a trial, the horrors of suspense, and the inevitable vicissitudes of passion, leave not the mind in a due disposition to the holy exercises of prayer and self-examination." The ignominious mode of death, and, above all, the publicity of the execution, greatly aggravated his sufferings: he would have reconciled himself more easily, he said, to his fate were he to be executed *privately*. This acknowledgment is of considerable importance, showing how much the publicity of the punishment increases its terror. So also when Henry Fauntleroy was found guilty of forgery, and condemned to death, the faculties of his mind were completely prostrated; indeed, his spirits fell, and he never rallied from the day of his apprehension. When the fatal hour arrived, he was so feeble and so exhausted, that the officials were under the necessity of supporting his tottering frame to the drop; and it is more than probable that had a reprieve then arrived, it would have been too late to save his life. A melancholy case, showing the fatal shock which the mind and nervous system may receive from violent mental emotion, under such distressing circumstances, was some years ago related in the House of Commons :—" Upon the home circuit, a young woman was tried for having stolen above forty shillings in a dwelling-house. It was her first offence, and was attended with many circumstances of extenuation. The prosecutor, as he stated, appeared from a sense of duty; the witnesses very reluctantly gave evidence, and the jury still more reluctantly their verdict of Guilty. It was impossible not to observe the interest excited in the court. The judge passed sentence of death. She instantly fell lifeless at the bar. Lord Kenyon, whose sensibility was not impaired by the sad duties of his office, cried out in great agitation from the bench, 'I don't mean to hang her ;—will nobody tell her I don't mean to hang her.' "* But it was too late ; she was already dead. " I have witnessed," says Sir Samuel Romilly, "the awful and heart-rending effects which the delivery of this sentence has had on criminals, and in some instances I have seen the judge send to the prisoners, such was their dangerous state, to assure them that sentence would not be executed."† Not many years ago a man of the name of May was indicted, along with Bishop and Williams, for the murder of an Italian boy, for the purpose of selling his remains to one of the anatomical schools. In consequence of the confession made by Bishop, after sentence of death had been pronounced, the Secretary of State sent a reprieve for May. Upon its communication to him, "his countenance assumed a livid paleness, the blood forsook his lips, his eyes appeared set, and the pulsation of the heart could scarcely be distinguished. All persons present thought he could not possibly survive, and that the warrant of mercy had proved his death blow. It was nearly a quarter of an hour before he was restored to his faculties."‡

While the mind is in some cases thus overwhelmed by sentence of death, in others it may remain obdurate, and almost insensible to its impending fate. One of the most atrocious social criminals whose deeds were ever recorded in the annals of crime was Burke, who was executed for murdering people and selling their dead bodies for dissection, in Edinburgh. This miscreant had so familiarized his mind to the atrocious crimes he perpetrated, that he witnessed the struggles of his dying victims with the utmost indifference. After his condemnation, he stated that he used frequently to think that the murders he committed would one day be discovered, and that when he went to bed at night, before going to sleep, he often reflected how

* House of Commons debate, 9th March, 1811.

† " The Speeches of Sir Samuel Romilly. Vol. i. p. 460. Ridgway. 1820.

‡ "Annual Register," December, 1831.

he should conduct himself on the scaffold. When the morning for the execution arrived, he expressed great anxiety that he should be well dressed, and borrowed from one of the turnkeys a suit of clothes, a pair of shoes, and a white pocket handkerchief, which he promised him to keep clean, and have returned to him after his death. The morning was extremely wet; the rain poured down in torrents; it was necessary for him to walk a short distance from his prison to the head of Libertyn's Wynd, where the scaffold was erected. Faithful to his promise, he picked his way on tiptoe over every prominent stone in the causeway, until he reached the platform. There, after the usual preliminaries had been gone through, he dropped the white handkerchief on the wet boards to kneel upon. Then, having said his prayers, he rose up: but the difficulty was to recover the handkerchief, for his arms were pinioned behind him. After several ineffectual attempts, he succeeded in picking it up, and handing it to one of the attendants, said, "Return this, along with the clothes I have on, to ——, the person from whom they were borrowed;" which were the last words, we believe, he uttered. We must, however, consider that in most cases where culprits have behaved on the scaffold with indecorous indifference and levity, they have been actuated by a desire of concealing or disguising their real feelings. Nor does it follow that they entertain less a dread of death.

We believe that in the majority of cases the mind receives a shock from which it seldom recovers; and the mental faculties are frequently so much impaired as to render the confession of such prisoners often incorrect; not that there may be any intention to deceive; but the mind in so disturbed a state cannot take a clear retrospect of past events. Hence we have heard that the governors and chaplains of Newgate seldom place much dependence upon the details of such confessions. Feuerbach and Heinroth, in Germany, who are high authorities in criminal jurisprudence, appear also to be of this opinion. In the case of the Mannings, it is clear that the husband so completely lost his self-possession, that the confessions he made piecemeal could not in all their details be relied upon; while the mental state of the wife was such as indicated a certain amount of aberration. No rational being would have manifested the excitement which it is notorious she did up to the very last hour. Her whole conduct during the interval between the sentence of death and execution manifested an abnormal state of mind. Her frequently indecent conversation,—her anxiety about her dress, and general inconsistency of conduct, evinced an amount of irrationality which must be obvious to every physician engaged in this department of the profession; but we by no means wish to imply that this state was sufficiently developed to justify so atrocious a criminal being exempted from capital punishment.

The shock which the mind in its healthy state receives when sentence of death is passed, and the effect of consequent despondency, is such as visibly to affect the nervous system. Professor Monro, in his "Elements of Anatomy," states that he found the brains of executed criminals considerably softer than is natural. The immediate effect of such a mental emotion as we are now considering is to depress or enfeeble the action of the heart. The respiration is difficult, because the blood is not propelled with sufficient force through the lungs. The usual quantity of blood circulating through the brain is diminished. Hence the mental perceptions become dull and confused. This supply of nervous energy usually diffused through the system fails; and there is consequently a rapid emaciation and gradual softening of all the tissues. Such are the very obvious psychological and physiological effects consequent upon the sentence of condemnation to capital punishment.

The effect of public executions on the popular mind next claims attention; whether from a principle of imitation, or by exciting a morbid desire for notoriety, such spectacles are presumed to have a most demoralizing tendency. They may certainly affect the imagination in a variety of ways. Horace Walpole, in his "Letters to the Countess of Ossory, mentions the case of a Mr. Fitzherbert, who went to see some convicts executed; and upon his return home committed suicide, by hanging himself in his own stable.* The effect of such spectacles upon the populace is curiously illustrated in the History of Denmark. "In the middle of last century," we are informed that "malefactors were attended from their prison to the place of execution by priests, and a numerous procession singing psalms, &c., which ended by a long discourse being addressed by the priest to the culprit, who was hung as soon as he had heard it. This spectacle, and all the pious cares bestowed upon the criminals, so far seduced the imagination of the common people, that many of them committed murder, purposely to enjoy such advantages; and the government was positively obliged to make hanging dull before it ceased to be an object of popular ambition."† "There can

* "Letters Addressed to the Countess of Ossory," by Horace Walpole. Lord Oxford. Vol. ii. Letter ii. London. 1848.

† "Tableau des Etats Danois," pas J. P. Catteau. 3 tom. 1802. Paris. See Edin. Rev. Vol. ii. 279. 1808.

be no doubt, observes an intelligent author, "but that the sight of state executions for supposed political or religious wrongs—such as the deaths of Charles I., the Marquis of Strafford, the Earl of Derwentwater, &c.—largely gained adherents to the cause for which those eminent persons suffered, and that the records of their deaths will always enlist the fervent sympathies of a generous people."*

To invest any form of public punishment with a *prestige* that may excite the compassion and sympathies of the multitude is unwise; but we must not confine our views of their effect to the ribald conduct of the assembled spectators; for however great may be the concourse of people, they form only a very insignificant section of the community. We must remember that the account of every execution is in a very few hours diffused, by means of the press, into multifarious channels, and read by two or three millions of persons; and who knows the salutary moral lesson which may from such details be gathered and inculcated in many a poor and humble family? We must not on this point form too hasty a judgment. It is true that robberies have been committed at the foot of the gallows; in every mob, pickpockets will be found; but it by no means follows that their evil propensities are called into existence by the sight they have assembled to witness. To obviate the supposed evils to which such spectacles give rise, it is proposed that executions shall in future take place privately, or within the precincts of the gaol. This would introduce a bastile principle into our social system, which we feel persuaded would be abhorrent to every English mind. The object of the *ultimum supplicium*, as Blackstone observes, is to deter others from perpetrating the like offences. The example, therefore, must be public; and when this can be dispensed with, it may fairly be presumed that capital punishments will be no longer necessary for the safety of our political or social state.

* "The Pencilwood Papers." Vol. ii. p. 31. London. 1846.

